# Green tea and tulsi extracts as efficient green corrosion inhibitor for aluminum alloy in alkaline medium

**DOI:** 10.1016/j.heliyon.2023.e16504

**Published:** 2023-05-29

**Authors:** Mohammad Asaduzzaman Chowdhury, Nayem Hossain, Md Mir Shakib Ahmed, Mohammad Aminul Islam, Safiul Islam, Md Masud Rana

**Affiliations:** a Department of Mechanical Engineering, Dhaka University of Engineering and Technology (DUET), Gazipur, 1707, Bangladesh; b Department of Mechanical Engineering, IUBAT-International University of Business Agriculture and Technology, Bangladesh; c Department of Mechanical Engineering, Dhaka University of Engineering and Technology, Gazipur, Bangladesh

**Keywords:** Tulsi extract, Green tea extract, Alkaline medium, Green corrosion inhibitor, Surface morphology

## Abstract

Corrosion is a major issue in every industrial system. As a result of its widespread application, aluminum suffers enormous annual losses due to corrosion. Scientists are continually on the lookout for effective anti-corrosion strategies. Corrosion may be reduced in a number of ways, but many of them are harmful to the environment, so it's important to find a green alternative. Corrosion inhibitors in aluminum alloys can be found in green tea and tulsi extract. In this research, we found that aluminum alloy 1100 (Al-1100) ina 10% NaOH solution was inhibited by both green tea and Tulsi extract. Samples of AL alloy are submerged in 10% NaOH solutions with and without an inhibitor for a total of 25 days. The weight-loss technique is used to determine the effectiveness of an inhibitor, with tulsi extract far outperforming green tea with the best efficiency of 83.93% compared to the greatest efficiency of 14.29% for green tea. After being submerged in an inhibitory solution, an aluminum alloy surface developed an adsorbed protective layer, which is chemical adsorption, as seen by FTIR (Fourier-Transform Infrared Spectroscopy) spectroscopy. Green inhibitors those are present on the surface of the aluminum alloys are less corrosive confirmed by the SEM (Scanning Electron Microscopy) analysis. The chemical particles were found to be present as a coating over AL alloy surfaces, as determined by EDS (Energy Dispersion Spectroscopy) testing. In a10% NaOH solution, Al-1100 is inhibited more effectively by tulsi extracts than by green tea extracts.

## 1 Introduction

Corrosion is the gradual weakening and disintegration of metal over time that results in the formation of rust. Using inhibitors is only one of several strategies for protecting against this harmful corrosion. Metal Corrosion inhibitors, which can be either organic or inorganic, are commonly used to prevent the alkaline solution from completely dissolving the protective oxide coating on the surface of aluminum. This is done to reduce the amount of metal lost and the amount of alkaline solution needed [[1], [2], [3], [4], [5], [6], [7], [8], [9], [10], [11], [12], [13]]. With so many critical uses, it's no surprise that research into aluminum's corrosion behavior in a variety of hostile situations has maintained a high profile. Aluminum's resistance to corrosion in different conditions is due to the creation of a dense, cohesive passive oxide coating. Whenever the metal is exposed to powerful bases, this amphoteric surface coating degrades significantly [14].

Aluminum and its alloys have several uses in fields as diverse as transportation, aircraft, building, and electricity production [15]. Corrosion inhibitors, either organic or inorganic, are typically added to caustic media in industrial operations like alkaline washing, dissolving, and embossing, and to increase efficiency in devices like aluminum alkaline batteries [[2], [3], [4], [5], [6], [7], [8]]; however, a few of these inhibitors are poisonous, costly, and non-biodegradable [16].

Aluminum corrosion can be mitigated in an alkaline media with the use of inhibitors. Only a select few researchers have looked at the possibility of slowing aluminum's rate of self-corrosion by adding chemicals to an alkaline solution [[17], [18], [19], [20]]. Most of them are harmful to humans and animals, costly, and harmful to the environment. Therefore, it is important to create a corrosion inhibitor for aluminum alloys in an alkaline media that doesn't harm the environment. To that end, we settled on plant extracts since they are naturally occurring, inexpensive, and easy to extract. Extracts' phytochemicals, which are largely heterocyclic molecules, can interact strongly with the metal surface, preventing corrosion [21,22]. Plant extracts and chemical medications (drugs) are often used options to prevent corrosion that are safer for the environment [23,24]. Plant extract contains phytochemicals like alkaloids and flavonoids, which adsorb on metal surfaces and prevent corrosion because they include heteroatoms like N, S, O, and π -electrons, aromatic ring [15,[25], [26], [27], [28], [29]]. Aluminum and its alloys benefit from tea plant extracts' corrosion prevention properties [30]. Oleanolic acid, ursolic acid, rosmarinic acid, eugenol, carvacrol, linalool, and beta-caryophyllene are the primary chemical components of Tulsi, the majority of the carbon chain in the compounds is between C10 and C12, which has the highest IE%. Green tea also have some active constituent's but they don't have most carbon chain between 10 and 12, that's why they effect less than tulsi [31,32]. When comparing Tulsi and green tea, it's important to keep in mind that their chemistries are distinct enough that not all of their contents are involved for inhibition. Further detailed studies may be conducted to determine things like the precise absorption system of those compounds and the precise constituents responsible for inhibition, since it was previously stated that lengthy carbon chains inhibit better. In such case, we may make better use of plant active components in our fight against corrosion [33]. All sorts of advanced technologies, from space travel and alternative energy to electronics and more, rely on aluminum's versatility [[34], [35], [36]]. Most aluminum alloys offer impressive corrosion resistance to the elements and other factors because they are coated with a biological oxide layer of around 5 nm in thickness. Strong acids and bases, however, quickly dissolve oxide coatings. Degradation of the protective layer allows chloride and other hostile ions to penetrate and begin the corrosion process locally [37]. Even though aluminum alloy 1100 has a high impurity content, it is frequently used in these applications. Al alloy 1100 showed greater resistance to pitting corrosion in saltwater, as measured by positive pitting potential values between 23° and 60° [38].

The novelty of this research work is that no other research used both tea and Tulsi as green inhibitor against aluminum alloy in alkaline medium. The purpose of the current study is to compare the effectiveness of leaf extracts like tea and tulsi in preventing the corrosion of Aluminum Alloys in 10% NaOH solution. Plant extracts' corrosion-inhibiting efficacy has primarily been studied utilizing fundamental electrochemical methods in a controlled atmosphere [39]. A recent study shows Corrosion may be reduced by 71.43% when green tea extract is used in the weight loss strategy, and by 85.711% when tulsi extract is used instead. In 10% H_2_SO_4_, tulsi extracts had a more noticeable anti-growth impact than green tea extracts [40]. The inhibitory performance was determined using weight loss and electrochemical corrosion tests. Besides, FTIR, SEM, PD and EDS analysis was performed to analyze the surface morphology.

## 2 Materials and methods

### 2.1 Material preparation

**Fig. 1:**
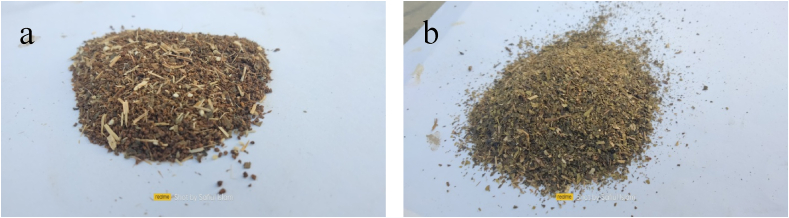
Crushed powder from the leaves (a) tulsi and (b) green tea. (For interpretation of the references to colour in this figure legend, the reader is referred to the Web version of this article.)

The sheets of Al-1100 alloy were purchased from Altech Aluminum in the Dhaka neighborhood of Gazipur. Plates of 1.5 by 1 by 0.2 cm were cut from clean, sanitized aluminum sheets. Distillation water is used to clean the surface, and then emery paper (P 600) is used to polish it. Elemental make up of Al-1100 by weight is 99.00–99.95% aluminum, 0.05–0.20% cooper, 0.95% (Max), 0.05% (Max) manganese, 0.95% (Max) silicon, 0.10% (Max) Zinc, 0.15% (Max) Residuals. Sodium hydroxide (10% NaOH) in concentrated form was procured from the regional chemical Store. Tulsi leaves were donated by the Agricultural Laboratory in the Department of Agriculture at International University of Business, Agriculture, and Technology (IUBAT). The green tea in this assortment is Ispahani Mirzapore, a well-known brand from Bangladesh. Extracts of Tulsi leaves and green tea can be seen in Fig. 1(a and b).

### 2.2 Leaves extract preparation

**Fig. 2:**
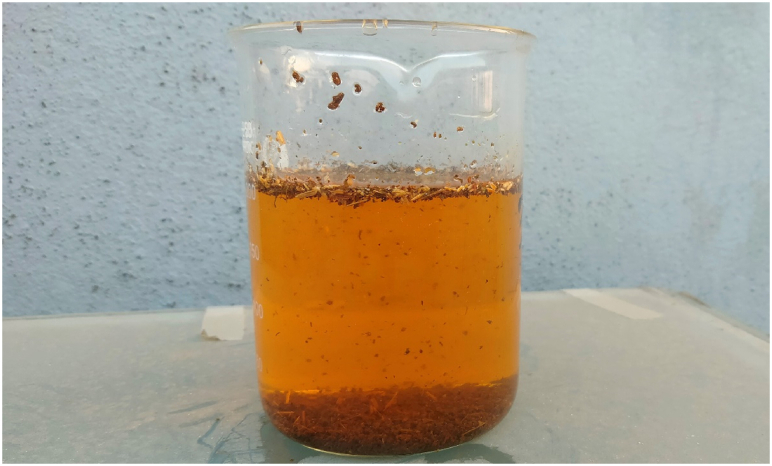
Prepared green solution. (For interpretation of the references to colour in this figure legend, the reader is referred to the Web version of this article.)

The NaOH that was obtained was in a solid state and was 10% NaOH. An end result pH of 11.51 was achieved by adding NaOH to100 ml of water. Tulsi leaves and green tea were thoroughly cleansed with distilled water. In order to obtain the extract, the leaves were first dried, powdered, and then mixed. Finally, 8 g of tulsi was adjusted to a pH of 10.5 by combining it with 5 ml 10%NaOH solution and immediately after we immersed the Al alloy into the solution. In another solution mixture,8 g of green tea and 5 ml of 10% NaOH were combined to create a solution with a pH of 10.5. Green solution preparation is depicted in Fig. 2.

### 2.3 Weight loss measurement

Abrading the Al-1100 specimens with varying grits of emery paper (P 600) before cleaning them with acetone and drying them off. Accurate electric balances were used to assess the weight of every sample. After that, we watched the corrosion impact by submerging the samples in the as-prepared solutions. After 25 days, the samples were removed, washed in distilled water, dried, and weighed again to determine the weight loss (WL). Each experiment was performed five times and the average data was considered. Following equation was used to determine corrosion rate (W_Corr_) [41]:

(1) $$Wcorr=\frac{87.6\times W}{A\times T\times D}$$

where,

W= Weight Loss in grams.

A = Area of the Specimen.

T = Exposure Time.

D = Density of the Al-1100 in g/cm^3^

The formulas below were used to figure out just how effective the corrosion is [[42], [43], [44]]:

(2) $$\eta w\%=\frac{Wcorr-Wcorr\left(inh\right)}{Wcorr}\times 100$$

### 2.4 Electrochemical corrosion test

The electrochemical measurements have been conducted in a 2-electrode electrochemical cell where an aluminum alloy sheet was used as a working electrode (WE) where the effective electrode area was 1 cm^2^ and a carbon rod as a reference electrode (RE)/counter electrode (CE). The electrochemical cell was filled ∼100 ml (electrolyte and/or electrolyte + corrosion inhibitor). The electrochemical test was performed for 3600 S where the working electrode was submerged in the solutions with or without inhibitors. The electrochemical workstation used is the Autolab PGSTAT204 coupled with a frequency response analyzer (FRA) 100 microHz to 1 MHz.

For testing electrochemical corrosion, both EIS and linear polarization experiments were conducted with respect to the OCP (open circuit potential). For EIS, a 10 mV AC amplitude and a frequency range of 10 Hz to 10 k Hz were used. For linear polarization, a scanning potential −0.1 to +0.1 vs OCP was used at a scan rate of 1 mV/s. The corrosion inhibition % was calculated using the following formula:

(3) $$I\%=\frac{Icorr-Icorr\left(inh\right)}{Icorr}\times 100$$

Here,

I% = Inhibition efficiency.

Icorr (inh) = Corrosion inhibition with inhibitor.

Icorr = Corrosion inhibition without inhibitor.

### 2.5 Characterization

#### 2.5.1 FTIR test

Fourier Transformed Infrared Spectroscopy (FTIR) analysis revealed that the corroded aluminum alloy surface included a variety of components. FTIR from PerkinElmer was used to conduct the study in the wavelength range of 500–4000 cm^−1^. This method is predicated on the observation that any infrared light that is taken in by a sample will, in due course, be transformed into heat. The values of the sample's absorption and transmittance might be utilized, in conjunction with the spectral data, to ascertain the sample's identification. FTIR is a technology that is non-invasive, adaptable, significant, and relatively straightforward for researching the effect of plant extracts on inhibiting properties [45].

#### 2.5.2 Morphological test

Micro/nano surface morphology of particles is often studied with the scanning electron microscope (SEM). One of the main advantages of the scanning electron microscope is that it can distinguish between particles smaller than 10 nm [45]. The eroded surface's morphology was studied using an EDS-enabled scanning electron microscope. In order to conduct the morphological test, a sample measuring 1.5by1by0.2 Cm was cut. The 20 kV acceleration voltage was used in the testing. Energy-dispersion spectroscopy's (EDS) significance It is widely recognized by researchers that characterization may be used to determine which components are present in a given sample. Since each element has a distinct atomic structure and hence creates a distinctive collection of peaks in the X-ray spectrum, the elemental makeup of any given sample may be determined from the spectrum [46,47]. Several conditions, including 10% NaOH, Tulsi extract, and green tea extract solutions, are tried out in order to ensure that the SEM and EDS studies work reliably under a wide range of conditions. Different quality images were collected to allow for in-depth examination.

## 3 Results and discussion

### 3.1 Weight loss analysis

**Fig. 3:**
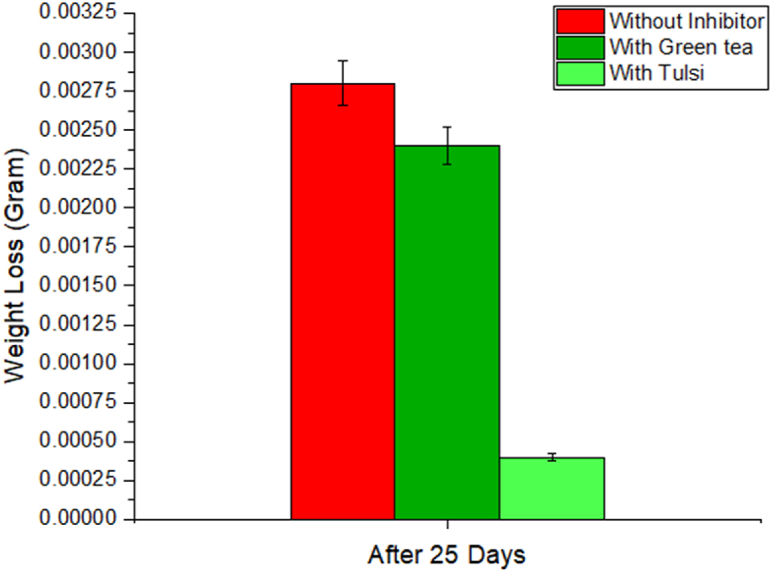
Use of inhibited solution to reduce corrosion.

**Fig. 4:**
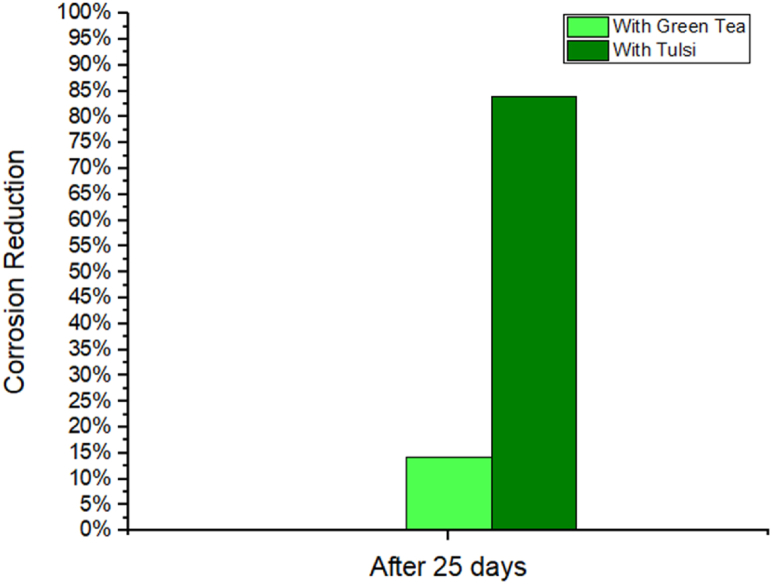
Bar Diagram of Corrosion Reduction in 10% NaOH medium.

Corrosion reduction comparison is shown by Fig. 3, Fig. 4. The corrosion rate is determined by taking the difference in weight between the pre- and post-corrosion weights of each individual Al-1100 sample and using a digital scale to make the measurement. The corrosion rate is expressed as a percentage. We can determine the efficiency of the corrosion using the given equations [[42], [43], [44]]:

(4) $$\eta w\%=\frac{Wcorr-Wcorr\left(inh\right)}{Wcorr}\times 100$$

When submerged in 10% NaOH medium for 25 days, the degradation of Al-1100 is compared in Table 1 to that of tulshi and green tea extract. When compared to the loss of 0.0028 g seen when no inhibitor is applied, the results show 0.0025 and 0.00045 g of weight loss from the solution of green tea and tulshi extract respectively from the same period of time. In Fig. 3, Fig. 4, a bar chart displays the outcomes of comparing weight loss and corrosion reduction. Significant corrosion reduction is obtained from tulshi and green tea. A comparison of the corrosion-inhibiting effects of green tea extract and tulsi extract reveals a 14.29% reduction for the first and an 83.93% reduction for the latter. Tulsi, when extracted in 10% NaOH, has a greater growth inhibitory impact than green tea, when extracted in the same solution.

**Table 1:** Weight loss and corrosion efficiency data comparison.

SL.		Al-1100's weight	Al-1100's weight after 25 days	Weight Difference	IE%
1	10% NaOH pH-11.51	3.335	3.3322	0.0028	–
2	10% NaOH at pH 10.5 with green tea extract	3.5349	3.5325	0.0024	14.28%
3	10% NaOH at pH 10.5 with Tulsi extract	3.6140	3.61355	0.00045	83.93%

### 3.2 Polarization analysis

**Fig. 5:**
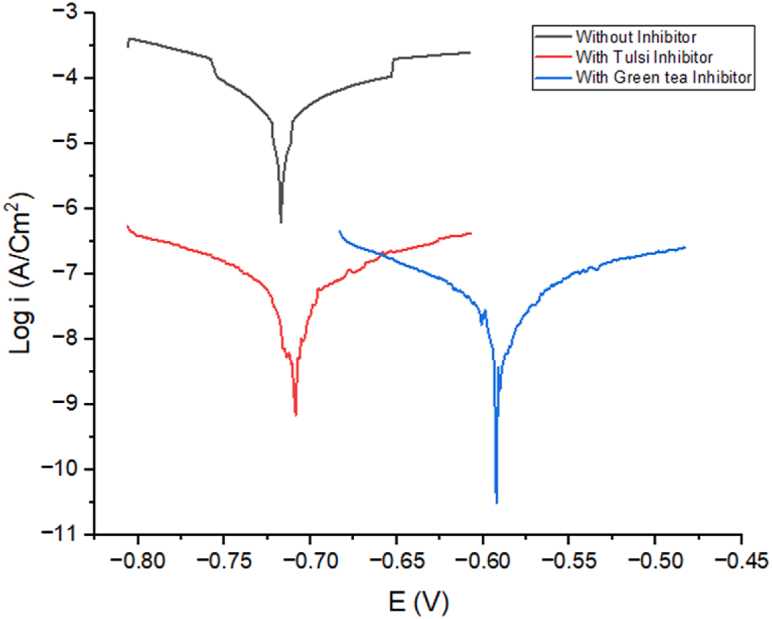
Analysis of Al-1100 using Tafel plots with and without tulsi and green inhibitor in a 10% NaOH solution. (For interpretation of the references to colour in this figure legend, the reader is referred to the Web version of this article.)

Tafel polarization data for Al-1100 in 10% NaOH solution at 303 K is shown by Fig. 5. Table 2 shows Tafel polarization parameter in the solutions. From the I_Corr_ values of these plots, we see that Green Tea was 80.51%efficient, whereas Tulsi was 46.16% effective. From the experimental data it can be said that Green tea is a better corrosion inhibitor compared to Tulsi tea. The chemical constituents of green tea made it a better corrosion inhibitor. When an inhibitor was present, the value of I_corr_ dropped dramatically compared to when none was present [40,[48], [49], [50]]. Table 3 shows the EIS parameters.

**Table 2:** Tafel polarization data at different solutions.

Sample Name	I_corr_ (A.cm^−2^)	E_corr_(V)	βa (V.dec^−1^)	βc (V.dec^−1^)	Corrosion Medium
Al-1100	−0.58064	−6.5498	2.8316	−2.8858	10% NaOH
Al-1100 with Tulsi extract	−0.97258	−7.1872	9.4718	−8.3803	10% NaOH
Al-1100 with green tea extract	−0.59571	−7.5838	20.025	−12.05	10% NaOH

**Table 3:** EIS parameters at different solutions.

Sample Name	R_ct_ (Ώ.cm^2^)	R_s_ (Ώ.cm^2^)	Corrosion Medium
Al-1100	5.43	1.15	10% NaOH
Al-1100 with Tulsi extract	45.96	1.59	10% NaOH
Al-1100 with green tea extract	7.25	1.21	10% NaOH

### 3.3 Electrical impedance spectroscopy analysis

As can be seen in Fig. 8, the measured values are consistent with the equivalent circuit. Solution resistance Rs, charge transfer resistance Rct, organic compound resistance Rmol at the interface, and constant phase element CPE make up the circuit. The constant phase element (CPE) is used to characterize the phase difference between an applied alternating current (AC) potential and the resulting current. Without any inhibitors present, the sample's EIS fitting curve is seen in Fig. 7 [51,52].

**Fig. 6:**
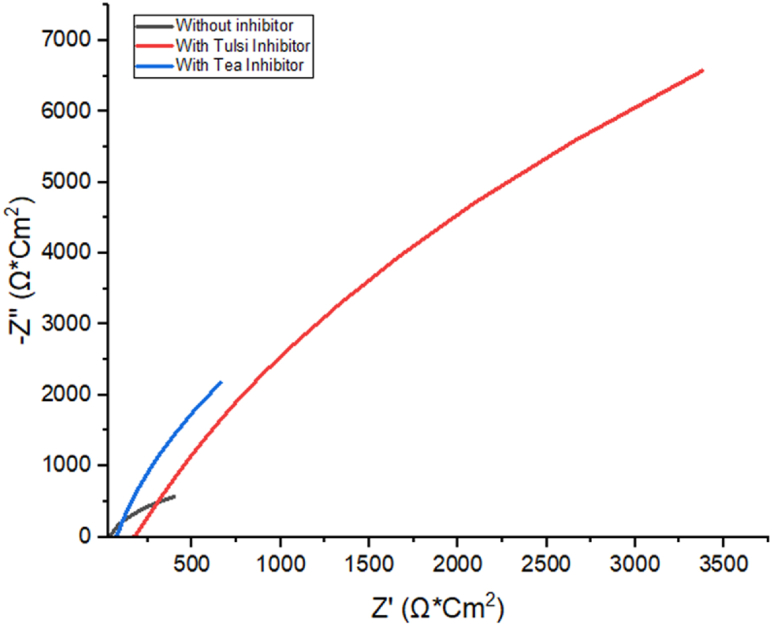
Samples of Al-1100 were submerged in 10% NaOH with or without extracts of the inhibitory compounds green tea and Tulsi and their respective Nyquist plots (EIS) were analyzed. (For interpretation of the references to colour in this figure legend, the reader is referred to the Web version of this article.)

EIS is an established method for studying the absorption mechanisms and corrosion behavior of aluminum alloys. The electrochemical characteristics of Al alloys were analyzed at 303 K in 10% NaOH solution both containing and without inhibitors. Nyquist plots were generated using the impedance data for Al alloys, and the results are shown as a line graph in Fig. 6. Tulsi and green tea extracts show the same behavior on Al alloys when introduced to a 10% NaOH solution. Observing the impedance map, one may see a gradually increasing curve. After being treated with green tea and Tulsi leaf extracts, the impedance property of the al alloys changed significantly. Higher impedance was measured at the highest inhibitor concentration because the bioactive components of the leaf extract were more readily adsorbed onto the Al alloy electrode [53]. As can be observed in the plot, the impedance of inhibitors derived from Tulsi extract is significantly greater than that of extracts derived from green tea leaves or of no inhibitors at all. Frequency dispersion describes this type of behavior, which may be linked back to the variations and hardness of solid substrates [42,54,55].

**Fig. 7:**
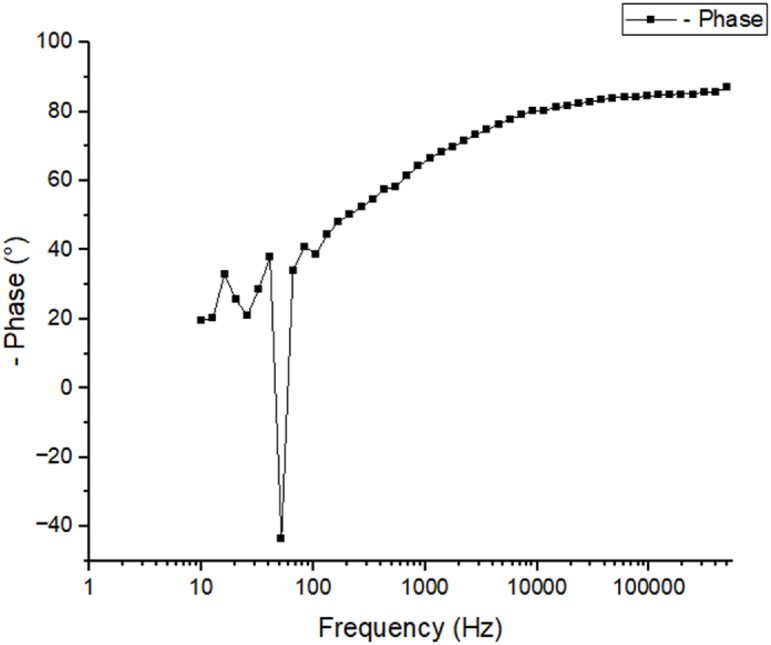
EIS fitting curve of sample in absence of inhibitors.

**Fig. 8:**
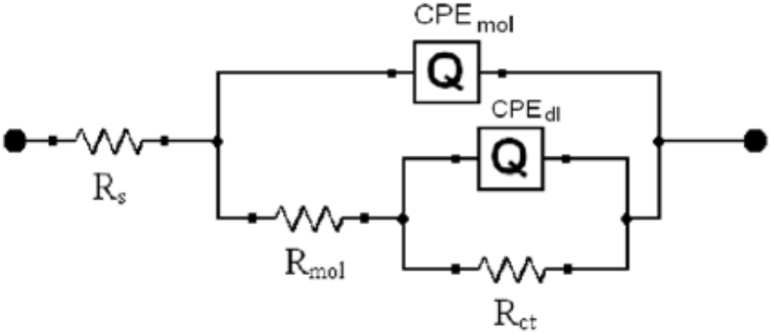
This study's electrochemical test's equivalent circuit.

### 3.4 FTIR analysis

**Fig. 9:**
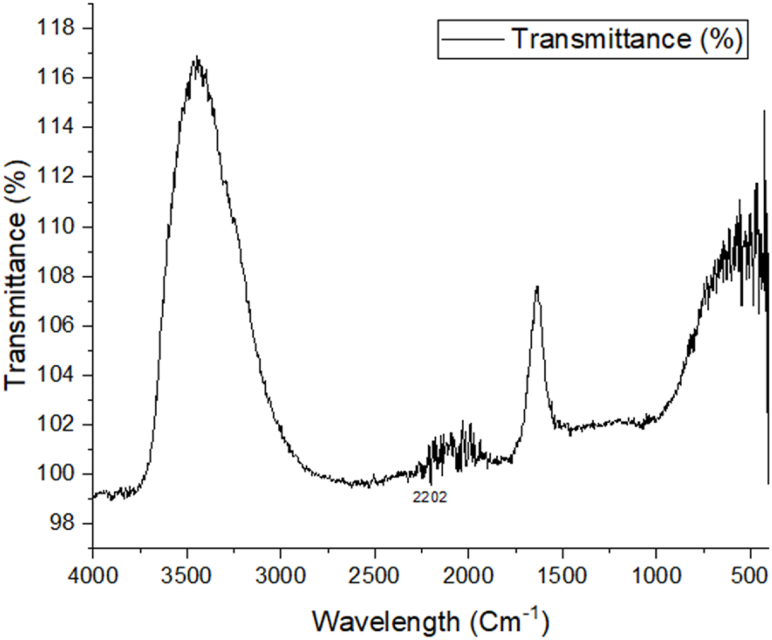
FTIR analysis of an AL alloy without inhibitor and immersed in 10% NaOH.

**Fig. 10:**
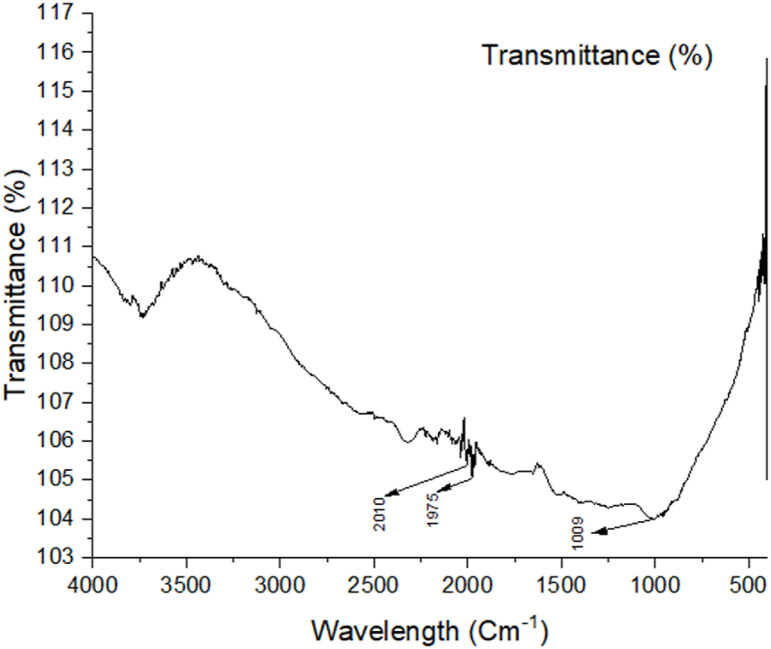
FTIR spectrum of the alloy submerged in the solution containing tulshi extract.

**Fig. 11:**
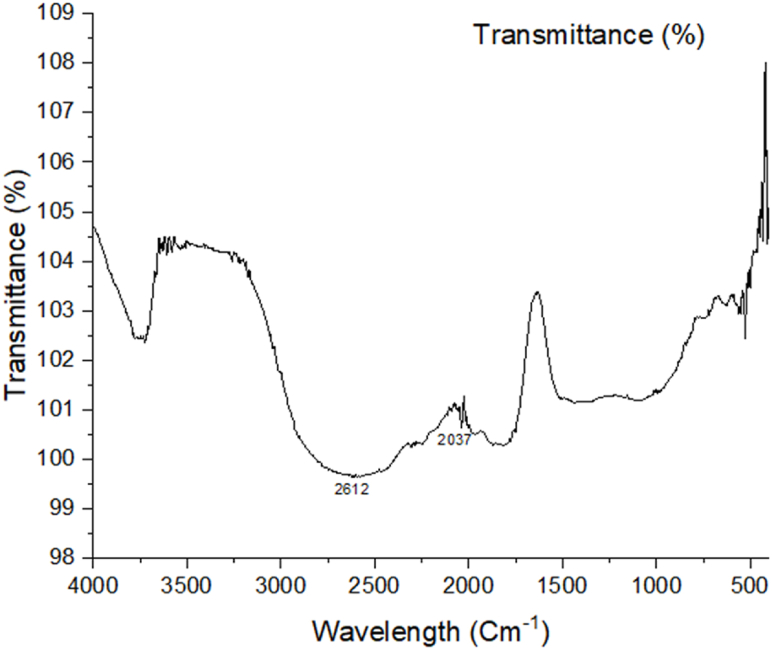
FTIR spectrum of the alloy submerged in the solution containing green tea.

The presence of heteroatoms (N and O), double bonds, and aromatic rings in organic molecules makes it easier for inhibitor molecules to become adsorbed on the surface of the metal. As a result, the amount of corrosion that occurs on the surface of the metals may be decreased [42]. Fig. 9, Fig. 10, Fig. 11 show the FTIR spectra of corroded aluminum samples in both uncontrolled and inhibited solutions, respectively. Because of the presence of heteroatoms (N and O), double bonds, and aromatic rings in organic molecules, the corrosion on the surface of mild steel may be decreased. These organic features help the inhibitor molecules get adsorbed on the metal surface, which in turn reduces corrosion. In Fig. 10, there is very minimum peak that can be seen where 2010 cm^−1^ shows N=C=S stretching, but in Fig. 11, which depicts the highly concentrated inhibited solution, there are peaks that can be seen that reflect the adsorption of medium-allene C=C=C in the range of 1976 cm^−1^. Isothiocyanate demonstrates a significant amount of N=C=S stretching in the 2037 cm^−1^ band as well. Unfortunately, in the FTIR analysis the transmittance is more than 100% which occurred due to some drift in the instrument. The reference spectrum was not recorded properly. This type of error is also seen in literature [49].

### 3.5 SEM analysis

**Fig. 12:**
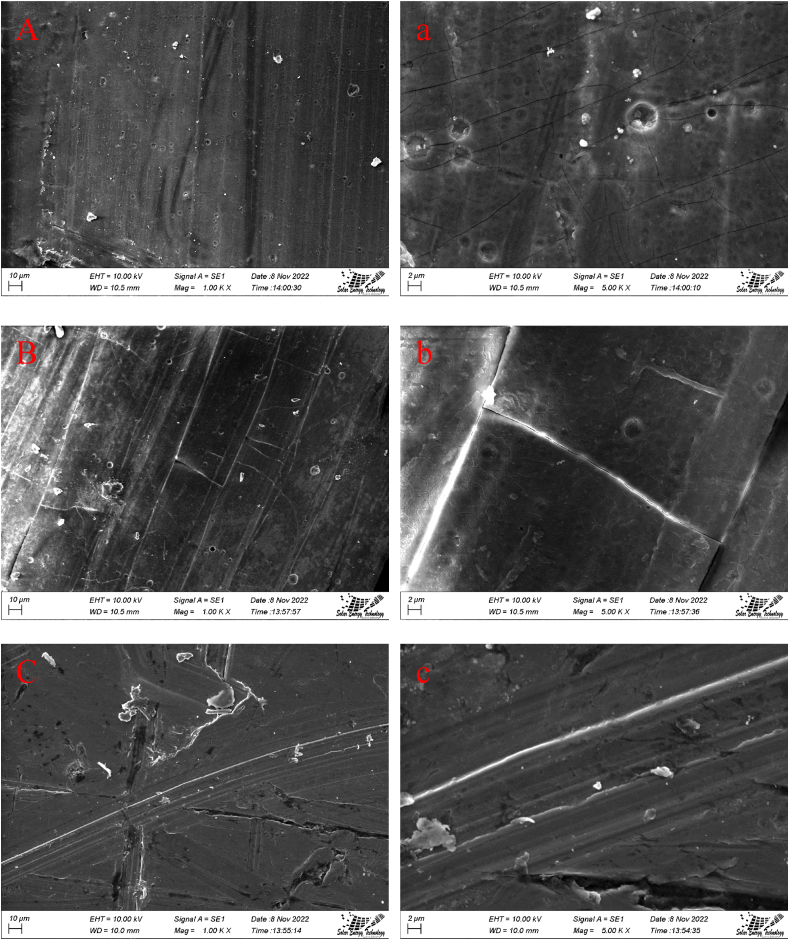
Sample after corrosion test in 10% NaOH solution at (A) 1.00 K X, (a) 5.00 K X,10% NaOH and green tea extract solution at (B) 1.00 K X, (b) 5.00 K X, 10% NaOH and tulshi tea extract solution at (C) 1.00 K X, (c) 5.00 K X. (For interpretation of the references to colour in this figure legend, the reader is referred to the Web version of this article.)

After being submerged in 10% NaOH solution for 25 days, an AL alloy samples characterized using SEM analyzer and shown by Fig. 12 (A, a, B, b, C, c). The figures confirm that the samples submerged in uninhibited solution are more damaged. This was the case despite the fact that the inhibited solution sample had been exposed to the same conditions. The pitting corrosion that has occurred on the surface of the aluminum alloy is depicted in Fig. 12 (A, a), where it can be seen that there is a substantial degree of the corrosion occurring. Besides, as can be seen in Fig. 12 (B, b) the surface of the Al alloy that was submerged in the inhibited solvent of green tea was extensively damaged by pitting corrosion and exhibited a significant amount of surface cracking. This was the case for the portion of the alloy that was examined. Green tea exhibited only 14.29% of corrosion inhibition which is the reason for the more corrosion on the surface of the alloy. The surface of the metal can be seen to have very few cracks in the scanning electron micrograph (SEM) image shown in Fig. 12 (C, c). This is because tulsi acted as a good corrosion inhibitor in the solution. The formation of a protective bio-film on the outer layer of the Al alloy contributed to the decreased rate of corrosion, which was connected to the fact that this mechanism was responsible for the reduction in corrosion [42,53,54]. The surface of the Al alloy will degrade more rapidly in the absence of these inhibitors. This is due to the fact that metals are easy to dissolve in alkaline settings [30,56,57].

### 3.6 EDS (Energy Dispersion Spectroscopy) analysis

**Fig. 13:**
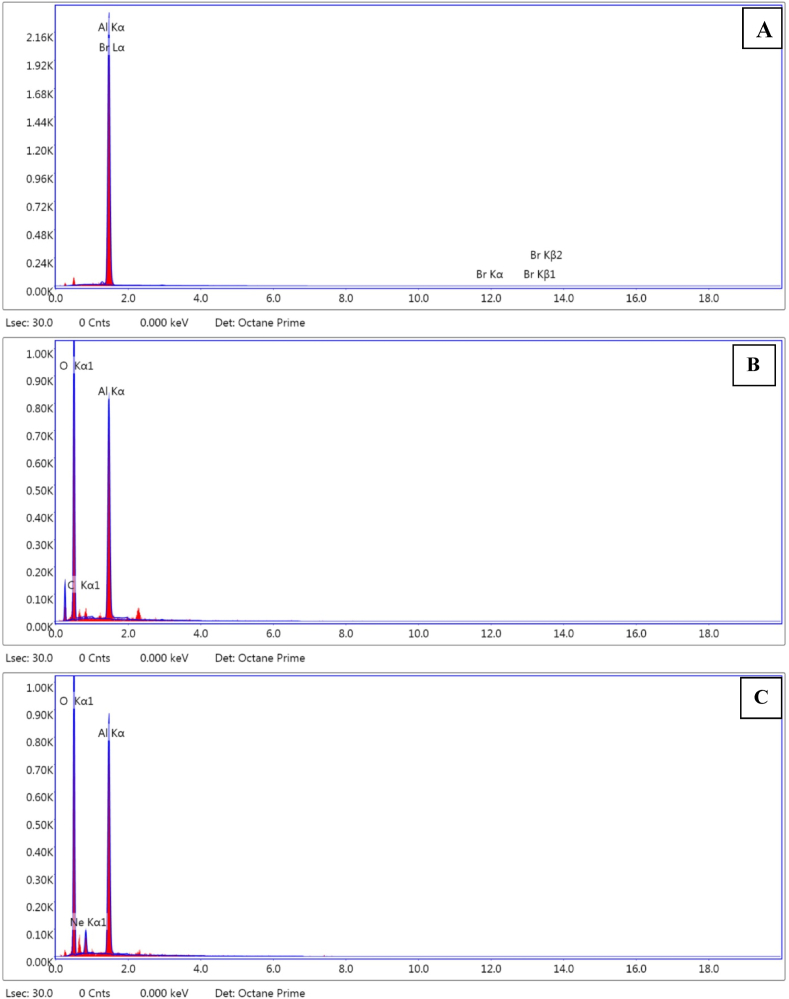
EDS analysis of the corroded samples at (a) 10% NaOH solution, (b) 10% NaOH and tulsi extract solution and (c) 10% NaOH and green tea extract solution. (For interpretation of the references to colour in this figure legend, the reader is referred to the Web version of this article.)

Fig. 13 (A), (B), and (C) show the results of a comparison between the EDS spectra acquired after corrosion test in the solutions, which was used to determine the aluminum's elemental composition. Three examples of EDS spectra are shown in Fig. 13: an Al alloy in 10% NaOH solution, a 10% NaOH solution with tulsi extract added, and a 10% NaOH solution with green tea extract added. EDS analysis shows that a large amount of aluminum and bromine is present on the surface when aluminum is immersed in 10% NaOH without inhibitors (Fig. 13(A)). When an Al alloy is immersed in 10% NaOH containing Tulsi extract, as shown in Fig. 13(B), the EDS spectrum shows substantially lower oxygen and higher percentages of Al which means less corrosion. Fig. 13(C) shows greater oxygen components in the EDS spectra after being submerged in 10% NaOH with green tea extract unlike Fig. 13(B). Tulshi extracts are more effective in corrosion reduction confirmed by the EDS spectra. During preparation of corrosive medium, some bromine may be there. Due to this unfortunate fact the reaction is occurred between bromine liquid and sodium hydroxide to form sodium bromate and water and the peak of bromine is appeared in EDS analysis. The contamination of bromine in solution is the limitation of this study, however in some cases it is difficult to avoid this situation especially in chemical process.

### 3.7 XRD (X-ray dispersion technique) analysis

**Fig. 14:**
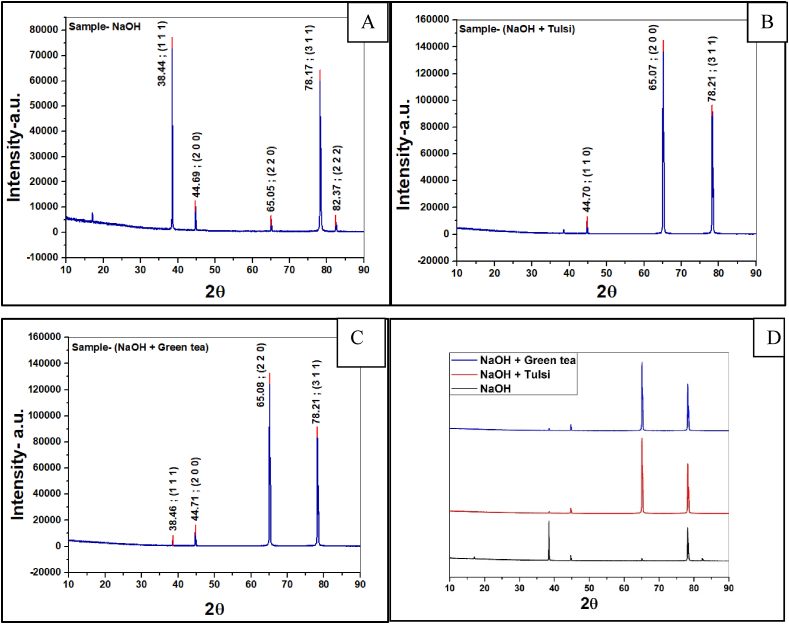
XRD graph of the alloy immerged in (A) 10% NaOH (B) Tulsi extracts solution, (C) green tea extracts solution and (D) combined graph. (For interpretation of the references to colour in this figure legend, the reader is referred to the Web version of this article.)

Fig. 14 (A-D) displays the results of an X-ray diffraction examination performed on 10% NaOH-medium-treated and untreated Al-1100. When seen at 2θ, the XRD peaks displayed a number of different index planes. The index planes at 38.44° are 1 1 1, 44.69° are 2 0 0, 78.17° are 3 1 1, and there are other small peaks, as shown in Fig. 14(A). Fig. 14(B) demonstrates that the addition of Tulsi extract inhibitor raises the intensity and lowers the peak crystallinity to 95.48% from 96.77% without inhibitor, while Fig. 14(C) demonstrates that the addition of green tea extract enhances the intensity, the index planes are nearly identical to those in Fig. 14(B), and crystallinity improves to 97.31%.

### 3.8 Mechanism of inhibition

The inhibitory effect of green tea and tulsi in a solution of sodium hydroxide can be described as follows based on the experimental and theoretical data obtained:

Green Tea + xOH_ ↔ [Green TeaOHx]^x-^

Tulsi + xOH^−^↔ [TulsiOHx]^x-^

The green tea and tulsi can be found as neutral molecules or as cations in aqueous alkaline solution. Two adsorption mechanisms can be considered usually. Water molecules are displace off the surface of the metal in chemisorptions method where electrons are exchanged between aluminum and oxygen atoms may be used to absorb the neutral green tea and tulsi on the metal surface. Depending on the recipient interactions between the aluminum d-orbitals that are unoccupied and the π-electrons of the heterocycle, the green tea and tulsi molecules could be adsorbed on the surface of the metal.

. Electrostatic interactions between the positively charged molecules and the deposited sulfate ions may also contribute to the adsorption of protonated green tea and protonated tulsi. As a result, the following might be a possible pathway for the formation of Al^3+^ metal complexes with Green Tea and Tulsi:

Tulsi + Al^3+^↔ [Tulsi– Al]^3+^

[TulsiOHx]^x−^ Al^3+^↔ [Tulsi–Al]^(3+X)+^

Green Tea + Al^3+^↔ [Green Tea– Al]^3+^

[Green TeaOHx]^x−^ Al^3+^↔ [Green Tea–Al]^(3+X)+^

In this research, these complexes may have been adsorbed on Al-1100 surfaces via van der Waals force to produce a protective coating that prevents corrosion [[50], [51], [52], [53]].

## 4 Conclusion

The purpose of this investigation and experimentation was to determine whether or not tulsi and green tea extracts might prevent the corrosion of Al-1100 in a 10% NaOH solution. Several characterization strategies are employed to examine the corroded state in this investigation. Using these several methods, we are able to examine the corroded state and inhibition efficacy from a variety of perspectives. According to our research using the WL technique, both green tea and tulsi extract are powerful corrosion inhibitors. Both the inhibitors reduced corrosion however the tulshi is far more effective at it, reducing it by 83.93% while the green tea only reduces it by 14.29%. When the extract was mixed the acid, tulshi had has a more potent growth-inhibiting action than green tea. Using tafel plots in alkaline medium, electrochemical study demonstrates that tulsi inhibits more effectively than green tea. Tulsi inhibitors are most effective in acidic environments, according to SEM pictures; they are also effective in alkaline media, but some pitting corrosion is observed. The SEM picture demonstrates that green tea has little inhibitory activity in both media. The XRD examination reveals that the Al-1100 treated with green tea has a higher degree of crystallinity than the same alloy treated with tulsi or left untreated. The findings from the FTIR spectrometer demonstrate that there are strong chemical bonds present on the surface of the metal with the inhibitor's green tea and Tulsi. After looking at the charts, graphs, and pictures that showed the data in more detail; it became clear that Tulsi inhibits aluminum in 10% NaOH solution better than green tea does.

## Author contribution statement

Mohammad Asaduzzaman Chowdhury: Conceived and designed the experiments.

Nayem Hossain: Conceived and designed the experiments; Wrote the paper.

Md. Mir Shakib Ahmed, Safiul Islam: Performed the experiments; Contributed reagents, materials, analysis tools or data.

Mohammad Aminul Islam: Analyzed and interpreted the data; Wrote the paper.

Md. Masud Rana: Analyzed and interpreted the data.

## Data availability statement

Data will be made available on request.

## Declaration of competing interest

The authors declare that they have no known competing financial interests or personal relationships that could have appeared to influence the work reported in this paper.
